# Bioinformatics and experimental approach reveal potential prognostic and immunological roles of key mitochondrial metabolism-related genes in cervical cancer

**DOI:** 10.3389/fonc.2025.1522910

**Published:** 2025-03-17

**Authors:** Qing Huang, Yang-feng Xu, Hui-ping Li, Ting Zhang

**Affiliations:** ^1^ Gynecology Department, Ningbo Medical Center Lihuili Hospital, Ningbo, Zhejiang, China; ^2^ Orthopedics Department, Ningbo Medical Center Lihuili Hospital, Ningbo, Zhejiang, China

**Keywords:** cervical cancer, mitochondrial metabolism, risk score, prognosis, immunity

## Abstract

**Background:**

Metabolic remodeling is the hallmark of cancer. In recent years, mitochondrial metabolism (MM) has been considered essential in tumorigenesis and cancer progression. Understanding the role of MM in cervical cancer (CC) can provide insights into disease progression and potential therapeutic targets.

**Methods:**

Clinical data of CC patients was downloaded from the UCSC Xena dataset, and differentially expressed genes (DEGs) were identified between tumor and normal samples. MM-related genes (MMRGs) were screened from the MSigDB database. DEGs and MMRGs were then intersected to identify differentially expressed MMRGs. A prognostic risk model was constructed based on these intersecting genes through Cox regression analysis, and its association with the tumor microenvironment and immune checkpoint-related genes was evaluated. Hub genes’ expression was evaluated in cells through qRT-PCR. Additionally, drug sensitivity analysis was conducted to explore potential therapeutic drugs.

**Results:**

We identified 259 overlapping genes between DEGs and MMRGs, with 55 being prognosis-related. Two molecular clusters were revealed, with C1 exhibiting poorer prognosis. A prognostic risk model comprising five genes (BDH1, MIR210, MSMO1, POLA1, and STARD3NL) was established, showing significant associations with survival outcomes of CC patients. Functional enrichment analysis revealed that DEGs between high- and low-risk groups were tightly associated with the immune system. Analysis of the immune microenvironment showed significant differences between different risk groups, with higher immune and ESTIMATE scores observed in the low-risk group. Additionally, expression levels of immune checkpoint-related genes were significantly correlated with the risk score. Drug sensitivity analysis identified potential therapeutic agents correlated with the expression of the five prognostic genes.

**Conclusion:**

Our findings underscore the importance of MM in CC progression and provide potential therapeutic targets for CC.

## 1 Introduction

Cervical cancer (CC) is one of the most prevalent gynecologic cancers, with an incidence of approximately 6.5% of all female cancer cases worldwide (1, 2). It is primarily caused by persistent infection with high-risk human papillomavirus (HPV) types (3). Despite advancements in screening programs and HPV vaccination efforts, CC remains a leading cause of cancer-related deaths among women globally (4). For patients with early or locally advanced CC, the 5-year survival rate exceeds 50% following surgical or chemoradiotherapy interventions (5, 6). However, the metastasis or recurrence significantly reduces survival rates, with a 5-year survival rate of only 10% for patients under such circumstances (4, 5). Therefore, it is urgent to delve deeper into the biological mechanisms of CC progression and identify novel prognostic biomarkers to enhance therapeutic strategies and patient outcomes.

Mitochondria, responsible for cellular energy generation, provide energy through the tricarboxylic acid (TCA) cycle and oxidative phosphorylation (OXPHOS) (7). Cellular energy imbalance is a recognized hallmark of cancer (8). In the 1920s, Otto Warburg postulated that cancer cells preferentially utilize glycolysis over OXPHOS for ATP production (9). For a long time, the major metabolic feature of tumor cells was considered to be the Warburg effect (10). However, in recent years, increasing evidence suggests that mitochondrial metabolism (MM) and function play a crucial role in tumorigenesis and cancer progression. Dysregulated mitochondrial function in cancer cells leads to alterations in energy production, metabolism, and redox balance, facilitating their proliferation, survival, and metastasis (11, 12). Moreover, mitochondria actively regulate apoptosis, allowing cancer cells to evade cell death signals and promote resistance to therapy (13–15). Therapeutic methods that target diverse pathways within MM, such as inhibiting cellular constituents engaged in mitochondrial synthesis, decreasing metabolite accumulation, or preventing energy production within mitochondria, have demonstrated therapeutic efficacy in various cancers (16–18). Several regents have been reported to alleviate CC by modulating MM. For example, Ginsenoside Rh2 induces CC cell apoptosis by suppressing mitochondrial electron transfer chain complex (19). Butyrate inhibited mitochondria-dependent apoptosis in CC cells (20). Therefore, MM could be a new therapeutic strategy for CC. Understanding the intricate interplay between MM and cancer biology holds promise for identifying novel therapeutic targets and developing personalized treatment strategies to combat CC.

This study aimed to elucidate the role of MM in CC progression and prognosis. Differentially expressed mitochondrial metabolism-related genes (MMRGs) in CC patients were identified and a prognostic risk model based on these genes was constructed. Through comprehensive bioinformatics analyses, we explored the clinical significance of MMRGs, as well as their association with tumor immune microenvironment and drug sensitivity, providing insights into potential therapeutic strategies for CC.

## 2 Methods

### 2.1 Acquirement of differentially expressed MMRGs

CC cohort was downloaded from the TCGA-UCSC Xena (http://xena.ucsc.edu). DEGs were identified between tumor samples (n = 305) and normal samples (n = 4) using the “limma” package in R software (version 4.1.3), with the criterion of |log2 fold change (FC)| > 1 and adjust p-value < 0.05. These DEGs were visualized in a volcano plot using “ggplot2”. MMRGs were acquired from the MSigDB database (gsea-msigdb.org). Overlap genes in the DEGs and MMRGs were visualized through “upsetR” and “VennDiagram” packages.

### 2.2 Functional enrichment analysis

Gene ontology (GO) and Kyoto Encyclopedia of Genes and Genomes (KEGG) enrichment analyses were performed using the “clusterProfiler”, with a criterion of p < 0.05. The results of GO and KEGG were visualized through the “Goplots” package in R.

### 2.3 Consensus clustering analysis

The MMRGs underwent univariate Cox regression analysis using the SPSS (p < 0.05). Consensus clustering analysis was then performed using R package ConsensusClusterPlus.

### 2.4 Establishment and evaluation of prognostic risk model

To mitigate overfitting, LASSO regression analysis was conducted on genes selected through univariate Cox regression utilizing the R package “glmnet”, with the penalty function lambda (0.5765) employed via cross-validation to identify and eliminate overfitting genes. Finally, a multivariate Cox regression analysis was performed on the retained genes using SPSS to establish a prognostic risk model for MMRGs in CC. The risk score was calculated using the obtained regression coefficients from the multivariate Cox regression analysis, with a formula as follows:


$$risk\;score=\sum_{i=1}^{n}exp_{RNAi}\;*\;Coef_{RNAi}$$

Subsequently, the risk score for each sample in the TCGA dataset was calculated. Based on their RiskScore values, samples were categorized into high- and low-risk groups, with the median RiskScore serving as the threshold. Kaplan-Meier (K-M) curves and receiver operating characteristic (ROC) curves were then generated for both groups using the R packages “survival” and “survminer” to assess the predictive performance of the model. The area under the ROC curve (AUC) for 1-, 3-, and 5-years overall survival was calculated. The expression of prognostic biomarkers in each sample was visualized in a heatmap using the “ggplot2” package in R.

### 2.5 Construction of nomogram

Clinical characteristics encompass age, TNM stage, and pathology stage. A nomogram was established based on these clinical features and risk scores. The calibration curve depicted the 45-degree dashed lines representing the best predictions of the nomogram. The nomogram and calibration curve was constructed using “rms”, “regplot”, and “survival” packages.

### 2.6 Evaluation of tumor immune microenvironment landscape

The stromal score, immune score, ESTIMATE score, and tumor purity were acquired using the ESTIMATE algorithm. They were then compared between different risk groups using the Wilcoxon rank-sum test. Immune cell abundance in the high- and low-risk groups was detected using the MCPcounter algorithm.

### 2.7 Immune checkpoint and immunotherapy response analysis

Immune checkpoint-related genes in CC were searched from the Checkpoint Therapeutic Target Database (CKTTD) (21). Expression levels of these genes in different risk groups were explored, and the correlation of them with risk score was visualized using the R package “ggplot2”. Tumor immune dysfunction and exclusion (TIDE) score was determined using the TIDE algorithm. p < 0.05 indicates statistical significance.

### 2.8 Drug sensitivity analysis

CellMiner (https://discover.nci.nih.gov/cellminer) encompasses 60 distinct cell lines originating from 9 types of malignancies, serving as essential screens in the development of novel anti-tumor medications. It includes 262 drugs, either FDA-approved or undergoing clinical trials (22). NCI60 drug response data were acquired from the CellMiner tool. Drug sensitivity between different risk groups was evaluated, and the association between drug sensitivity and prognostic gene expression was assessed using Pearson’s test.

### 2.9 Cell culture

Human cervical epithelial cells (Cat NO.: CP-H059) and cervical cancer cells Hela S3 (Cat NO.: CL-0350) were purchased from Wuhan Pricella Biotechnology Co., Ltd. All cells were cultured in a specialized medium containing Ham’s F-12K supplemented with 10% FBS and 1% P/S in an incubator at 37°C with 5% CO_2_. The medium was changed every 2–3 days.

### 2.10 Quantitative real-time (qRT)-PCR

Total RNA was extracted using TRIzol reagent (Invitrogen) according to the manufacturer’s protocol. The concentration and purity of the extracted RNA were assessed using a NanoDrop spectrophotometer (Thermo Fisher Scientific, CA, USA). The isolated RNA was then reverse-transcribed into cDNA with the PrimeScript RT reagent kit (Takara, Dalian, China). The reaction conditions were as follows: 42°C for 15 min and 95°C for 3 min. For quantification, qRT-PCR was performed using the Hieff UNICON Universal Blue qPCR SYBR Green Master Mix (Yeasen, Shanghai, China) on the ABI7900HT System. The thermocycling program was set as follows: initial denaturation at 95°C for 30 s, followed by 40 cycles of 95°C for 3 s and 60°C for 20 s. The relative mRNA expression levels were determined using the 2^-ΔΔCt^ method, with GAPDH serving as the internal control. The primer sequences were designed by Gene Creat Bioengineering CO. (Wuhan, China) and were shown in **Supplementary Table S1**.

### 2.11 Statistical analysis

Statistical analyses were conducted using R software version 4.2.0 and GraphPad Prism 8.0.2. Student t-test was used to compare the differences between the two groups, and the Pearson method was used for correlation analysis. All experiments were performed in triplicate, and data were analyzed using GraphPad Prism software. Statistical significance was determined using a Student’s t-test. Statistical significance was defined as p < 0.05.

## 3 Results

### 3.1 Identification of prognosis-related differentially expressed MMRGs in patients with CC

**Figure 1:**
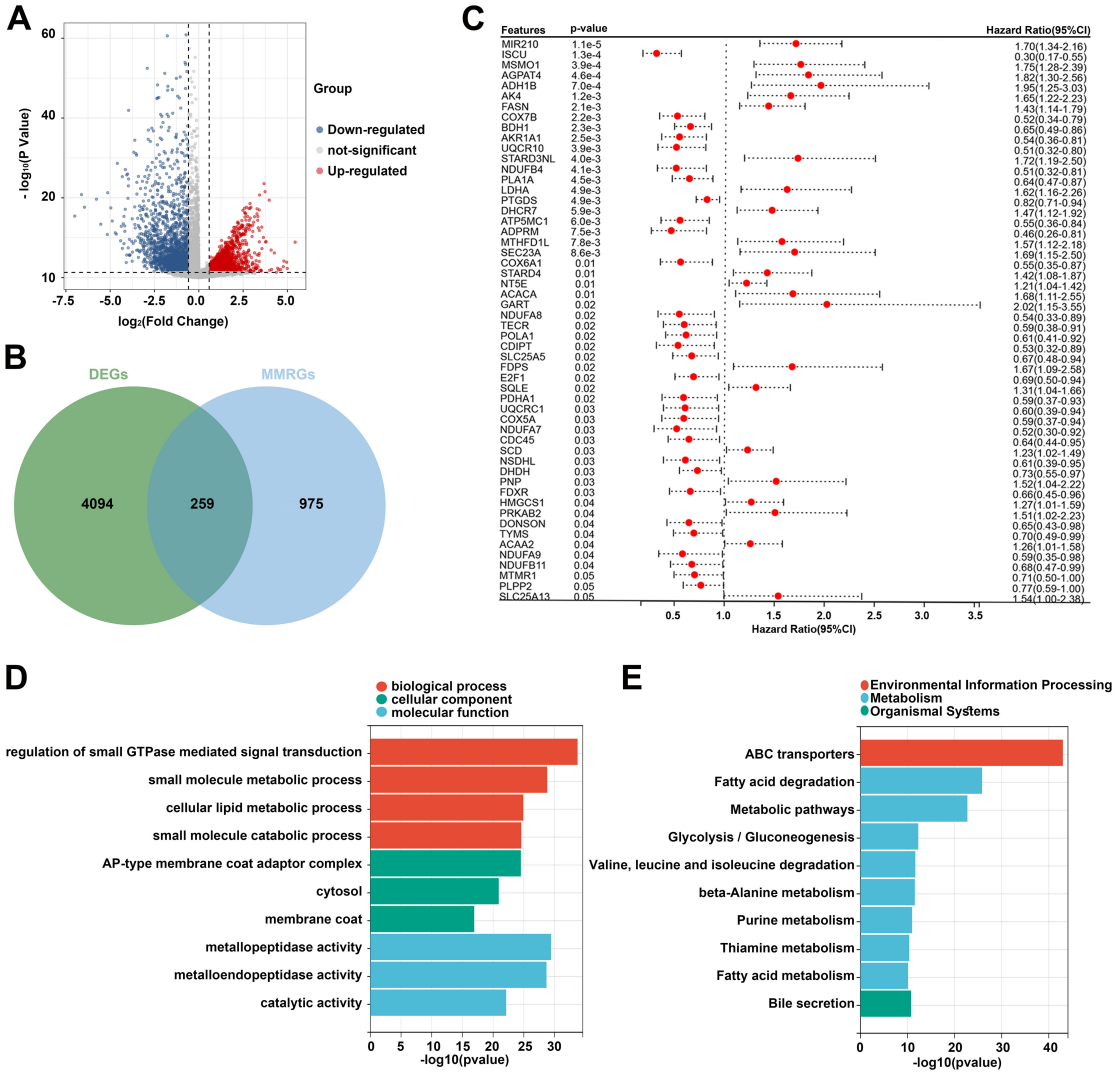
Identification of prognosis-related differentially expressed MMRGs in patients with CC. **(A)** Volcano plot of DEGs between tumor and normal samples in the TCGA database. The blue dots represent downregulated genes and the red dots represent upregulated genes. **(B)** A total of 4353 DEGs and 1234 MMRGs were screened from public databases, and 259 intersecting genes were identified between the DEGs and MMRGs using the Venn diagram. **(C)** Prognostic features were selected using the univariate Cox regression based on 259 differentially expressed MMRGs; p < 0.05. **(D, E)** GO and KEGG enrichment analysis was performed on the genes identified by univariate Cox regression analysis. CC, cervical cancer; DEG, differentially expressed gene; MMRG, mitochondrial metabolism-related gene; GO, Gene Ontology; KEGG, Kyoto Encyclopedia of Genes and Genomes.

Based on the TCGA dataset, 4353 DEGs were identified between the tumor and normal samples. Volcano plot showed 1976 upregulated genes and 2377 downregulated genes in the tumor group (**Figure 1A**). Additionally, 1234 MMRGs were screened from the MsigDB database (**Figure 1B**). Venn diagram revealed 259 overlapping genes in the DEGs and MMRGs (**Figure 1B**). Then, the overlapping genes were seeded on the univariate Cox regression, identifying 55 prognosis-related MMRGs (**Figure 1C**). GO enrichment analysis showed that these genes were associated with biological process, such as regulation of small GTPase mediated signal transduction, small molecule metabolic process, cellular lipid metabolic process, and small molecule catabolic process. They were also related to cellular components, including AP-type membrane coat adaptor complex, cytosol, and membrane coat. As for molecular functions, metallopeptidase activity, metalloendopeptidase activity, and catalytic activity were enriched (**Figure 1D**). The enriched KEGG pathways were related to environmental information processing, metabolism, and organismal systems, such as ABC transporters, fatty acid degradation, and bile secretion (**Figure 1E**).

### 3.2 Identification of two CC molecular subtypes

**Figure 2:**
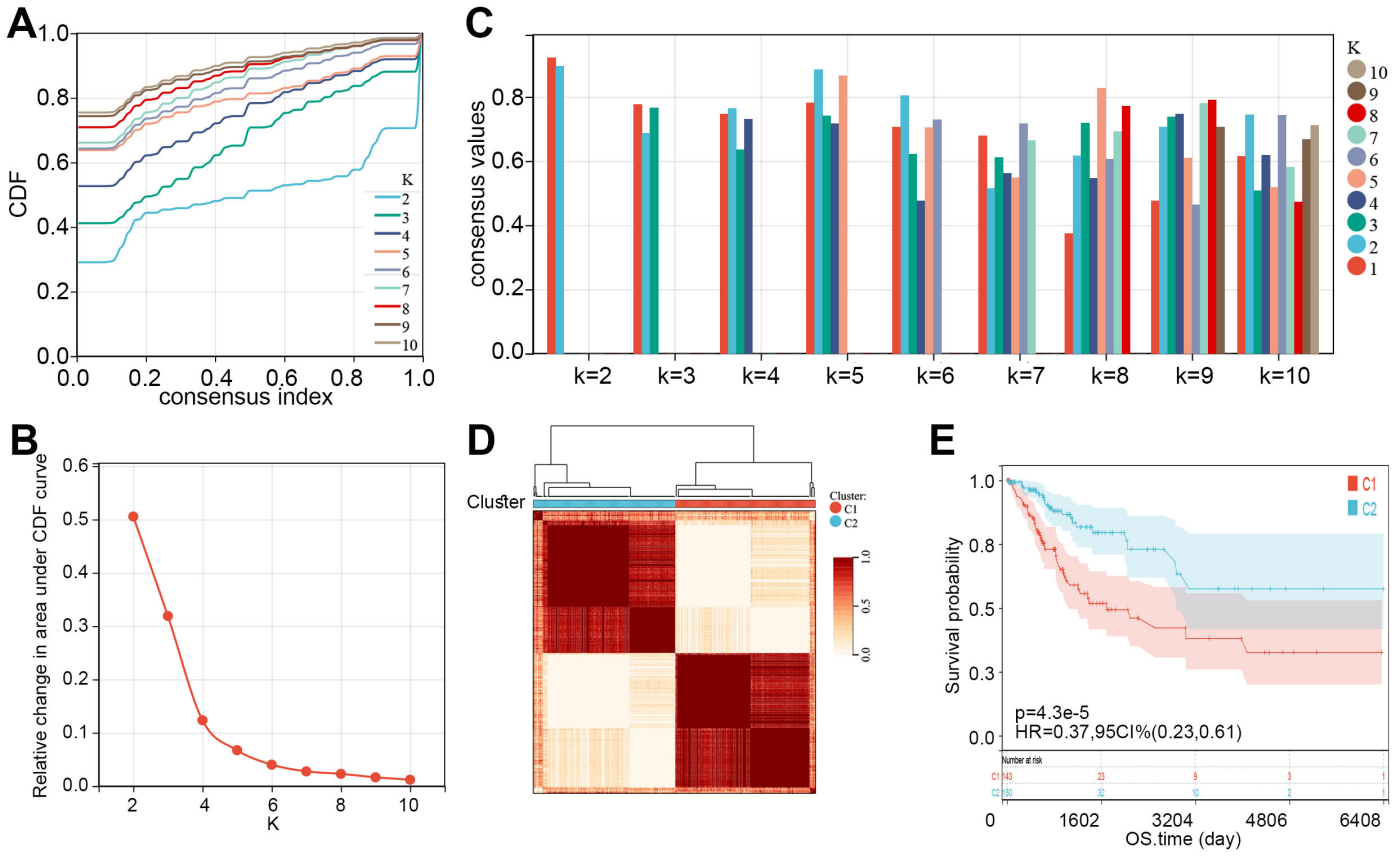
Two molecular subtypes of CC were identified. **(A)** Consensus CDF curves for different values of k (k = 2 to 10). **(B)** Relative change in the area under the CDF curve for different k values, indicating the optimal number of clusters. **(C)** Bar plot showing consensus values for different k values, with different colors representing varying cluster numbers. **(D)** Consensus clustering heatmap showing the division of samples into two clusters (C1 and C2) based on consensus matrix values. **(E)** K-M curve showed the overall survival time of CC patients between C1 and C2. CDF, cumulative distribution function; CC, cervical cancer; K-M, Kaplan-Meier; HR, hazard ratio; CI, confidence interval; OS, overall survival.

Subsequently, consensus clustering analysis was performed on the 55 prognosis-related MMRGs. The CDF curve showed that k = 2 is the optimal number of clusters (**Figures 2A, B**). **Figure 2C** displayed the consensus values for different k values, with the highest consensus value at k = 2. Therefore, all samples were clustered into two subtypes. The heatmap showed that the samples were well separated, with a clear distinction between the two subtypes (**Figure 2D**). K-M curves revealed that patients in C1 had significantly poorer OS than those in C2 (**Figure 2E**). Compared to C2, patients in C1 had higher pathological stages (**Table 1**). These results suggest that patients in C1 may have a worse prognosis and a higher degree of malignancy.

**Table 1:** Differences in stages and grades between two different subtypes.

Variables	Missing	Category	Total (n=293)	C1 (n=143)	C2 (n=150)	p
Stage, n(%)	6	I	158(55.052)	66(47.482)	92(62.162)	**0.029**
		II	65(22.648)	35(25.180)	30(20.270)	
		III	42(14.634)	22(15.827)	20(13.514)	
		IV	22(7.666)	16(11.511)	6(4.054)	
Grade, n(%)	28	1	19(7.170)	9(7.317)	10(7.042)	0.709
		2	129(48.679)	63(51.220)	66(46.479)	
		3	117(44.151)	51(41.463)	66(46.479)	

**Figure 3:**
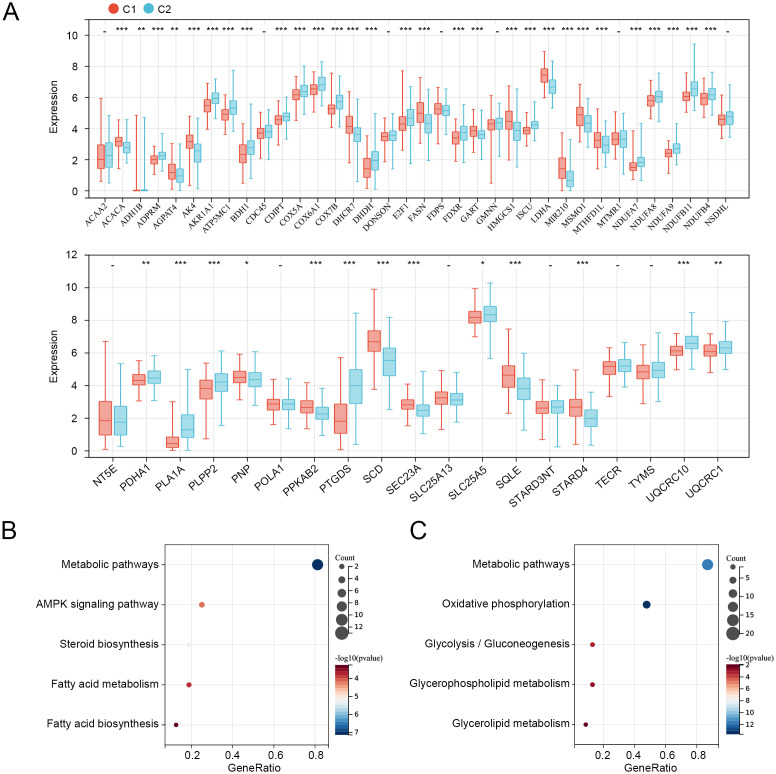
Expression and functions of prognosis-related genes in C1 and C2. **(A)** Expression levels of 55 prognosis-related genes in C1 and C2. **(B)** KEGG pathways related to overexpressed genes in C1. **(C)** KEGG pathways related to downregulated genes in C1. *p < 0.05, **p < 0.01, ***p < 0.001, “-”means no significant.

We then compared the expression levels of 55 prognosis-related MMRGs between C1 and C2. As shown in **Figure 3A**, 17 MMRGs were significantly overexpressed and 25 MMRGs were downregulated in C1. KEGG pathways analysis revealed that both overexpressed genes and downregulated genes in C1 were associated with metabolic pathways (**Figures 3B, C**). These results indicated that metabolic pathways may be involved in the imbalance of these differentially expressed prognosis-related genes to regulate the CC tumor microenvironment and progression.

### 3.3 Establishment of the MM-related risk model for patients with CC

**Figure 4:**
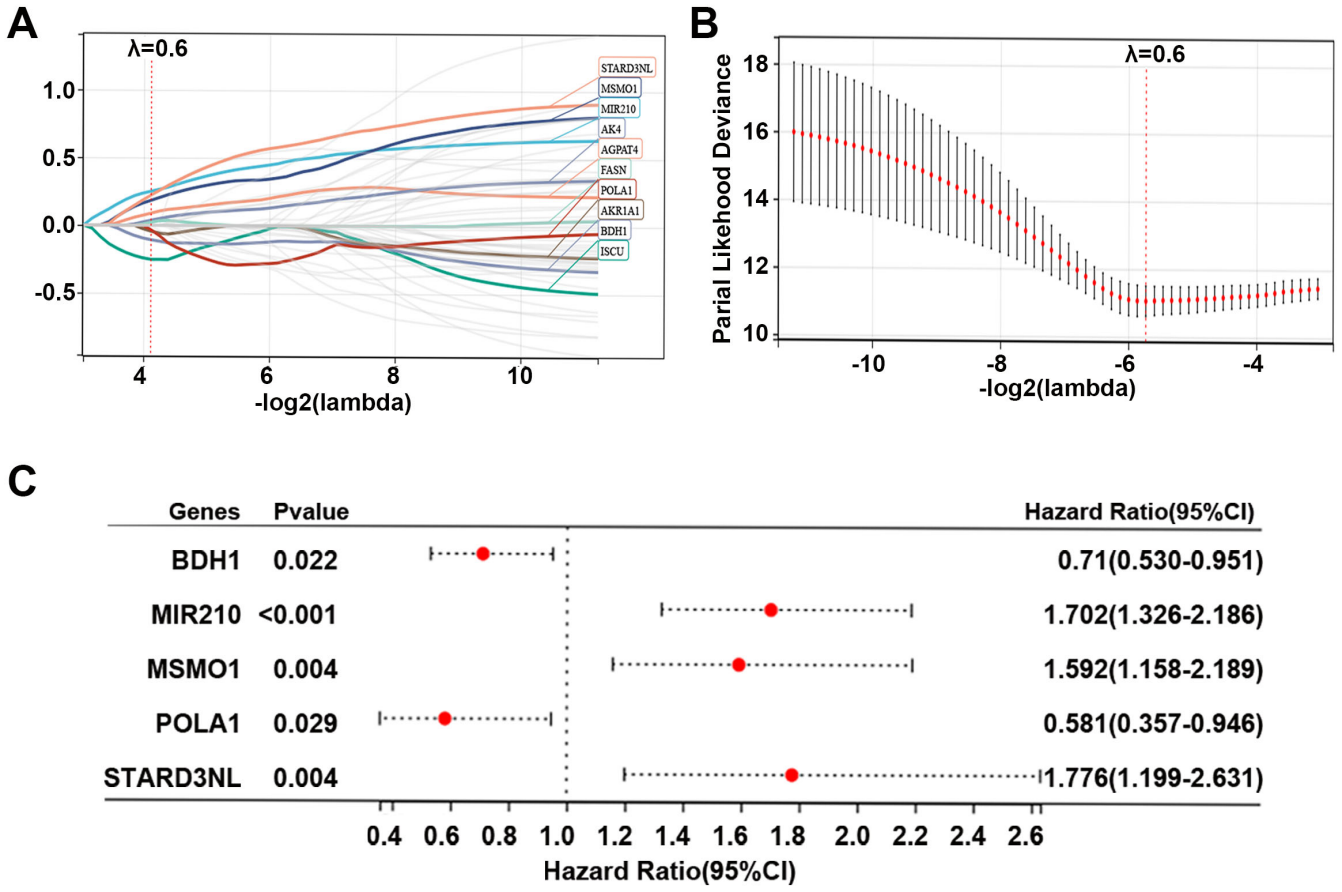
Identification of prognostic genes in CC. **(A, B)** Lasso Cox regression identified 10 genes related to the prognosis of patients with CC, and 10-round cross-validation was performed to detect the optimal values of the penalty parameter. **(C)** Multivariate Cox regression identified 5 prognostic genes based on the above 10 genes; p < 0.05. CC, cervical cancer; MMRG, mitochondrial metabolism-related gene.

To further explore the role of 55 MMRGs in CC prognosis, lasso regression was performed on these genes, and 10 MMRGs were identified (**Figure 4A**). The 10-round cross-validation was used to determine the optimal values of the penalty parameter (**Figure 4B**). Finally, using multivariate Cox regression, five genes, including BDH1, MIR210, MSMO1, POLA1, and STARD3NL were identified (p < 0.05, **Figure 4C**). Results revealed that BDH1 (HR = 0.71, 95%CI: 0.530-0.951) and POLA1 (HR = 0.581, 95%CI: 0.357-0.946) were protective features, while MIR210 (HR = 1.702, 95%CI: 1.326-2.186), MSMO1 (HR = 1.592, 95%CI: 1.158-2.189), and STARD3NL (HR = 1.776, 95%CI: 1.199-2.631) were harmful features (**Figure 4C**).

**Figure 5:**
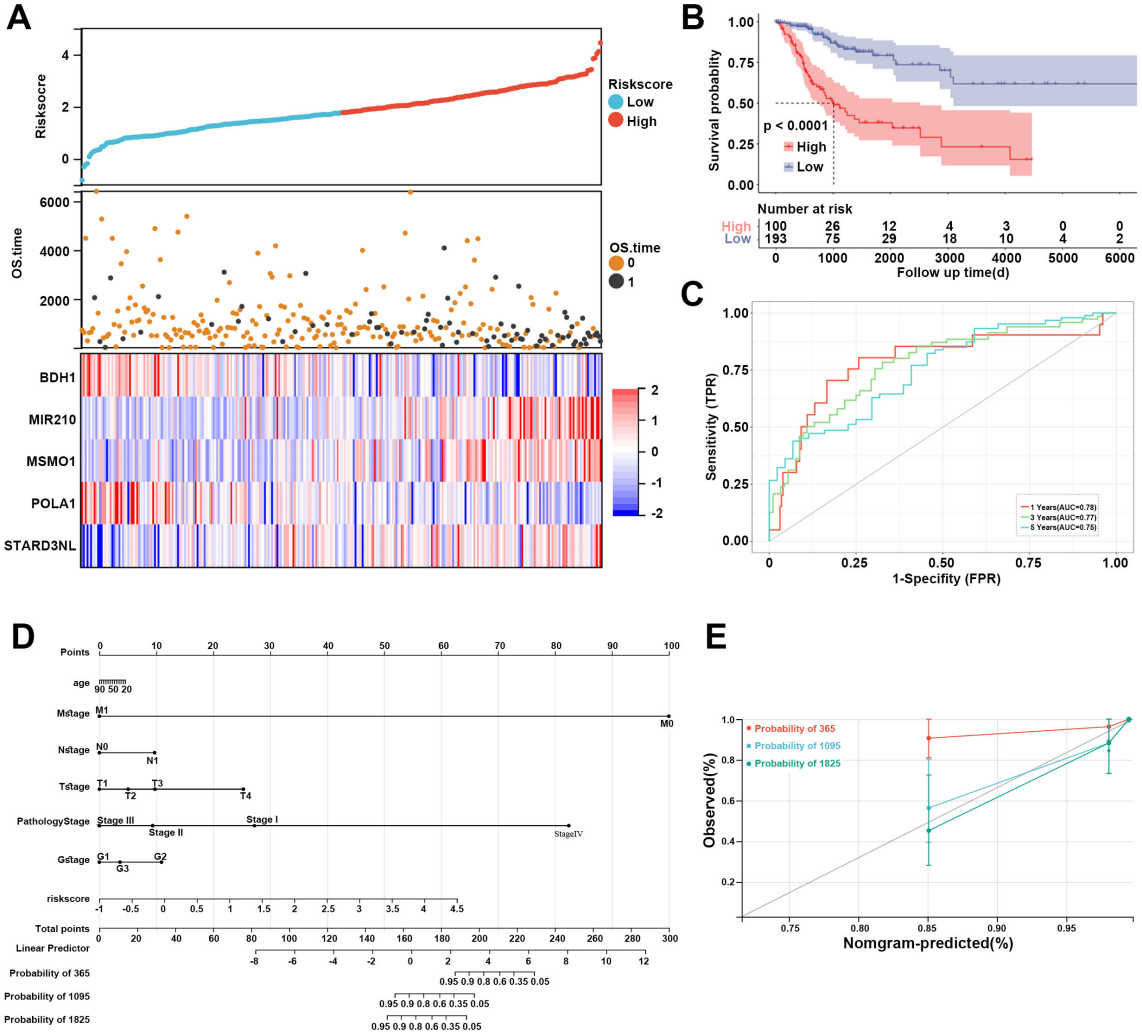
Assessment of MM-related risk model for CC. **(A)** Risk score distribution, OS time of each patient in the TCGA cohort, and heatmap of the five prognostic genes. **(B)** Kaplan-Meier curve revealed the survival probability of patients in different risk groups. **(C)** The ROC curve of the risk model at 1-, 3-, and 5-year. Red represents 1-year, green represents 3-year, and blue represents 5-year. **(D)** Nomogram for predicting 1-, 3-, and 5-year OS time for CC patients in the TCGA database. **(E)** Calibration curves of the nomogram observed 1-, 3-, and 5-year outcomes. MM, mitochondrial metabolism; CC, cervical cancer; OS, overall survival; ROC, receiver operating characteristic.

Furthermore, based on the expression levels of the above 5 prognostic genes, the risk score of each sample in the TCGA dataset was calculated as follows: risk score = -0.343*BDH1 + 0.532*MIR20 + 0.465*MSMO1 + -0.543*POLA1 + 0.575*STARD3NL. CC patients in the TCGA dataset were divided into high- and low-risk groups based on the median risk score. The risk score distribution of patients was visualized in **Figure 5A**. Our data indicated that patients with high risk scores had worse prognoses (**Figure 5A**). The expression of BDH1 and POLA1 was higher in the low-risk group, while the expression of MIR210, MSMO1, and STARD3NL was higher in the high-risk group (**Figure 5A**). The K-M survival curve demonstrated superior survival outcomes for the low-risk group compared to the high-risk group (**Figure 5B**). The AUCs of the enrolled patients at 1-, 3-, and 5-year were 0.78, 0.77, and 0.75, respectively (**Figure 5C**).

A nomogram was then constructed to show the performance of risk score and clinical features on CC prognosis. As shown in **Figure 5D**, the M stage, pathology stage, and risk score ranked in the top three in terms of contribution to predicting CC, followed by T stage, G stage, N stage, and age. The calibration curves of the nomogram for the probability at 3- and 5-year indicated a good clinical value (**Figure 5E**).

### 3.4 Functional enrichment analysis of DEGs between high-risk and low-risk groups

**Figure 6:**
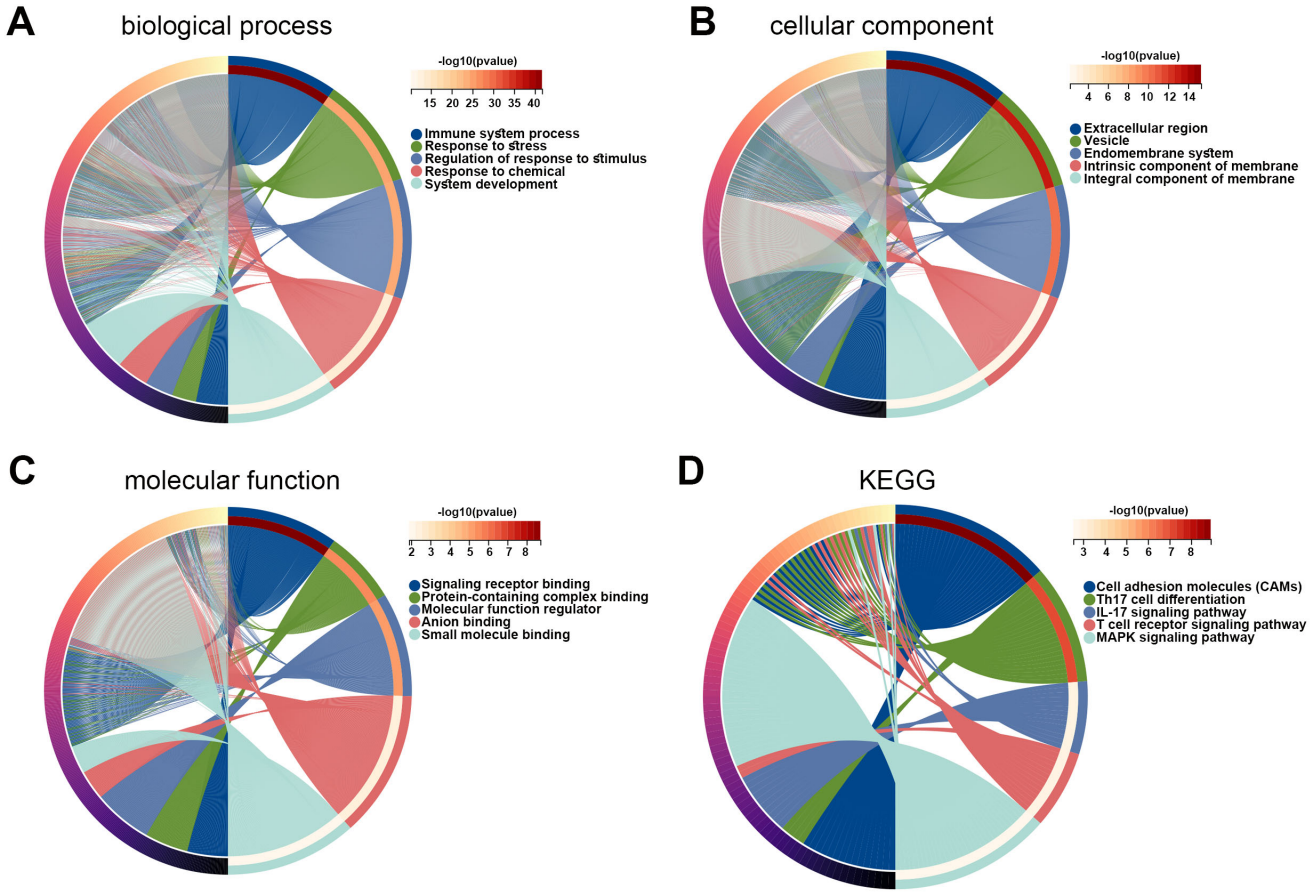
Functions of DEGs between high and low risk groups. **(A–C)** GO enrichment analysis was performed on DEGs in high and low risk groups, including biological process **(A)**, cellular component **(B)**, and molecular function **(C)**. **(D)** KEGG pathways related to DEGs in high and low risk groups. DEG, differentially expressed gene; GO, Gene Ontology; KEGG, Kyoto Encyclopedia of Genes and Genomes.

Subsequently, DEGs between high- and low-risk groups were identified, and the function of these genes was explored using GO and KEGG enrichment analyses. The top five enriched GO biological process terms were immune system process, response to stress, regulation of response to stimulus, response to chemical, and system development (**Figure 6A**). The top five GO cellular component terms included extracellular region, vesicle, endomembrane system, intrinsic component of membrane, and integral component of membrane (**Figure 6B**). The GO molecular function terms were mainly enriched in signaling receptor binding, protein-containing complex binding, molecular function regulator, anion binding, and small molecule binding (**Figure 6C**). The KEGG pathways were major enriched in cell adhesion molecules, Th17 cell differentiation, IL-17 signaling pathway, T cell receptor signaling pathway, and MAPK signaling pathway (**Figure 6D**). These results revealed that DEGs between high- and low-risk cohorts were tightly associated with tumor immune microenvironment.

### 3.5 Tumor immune microenvironment analysis

**Figure 7:**
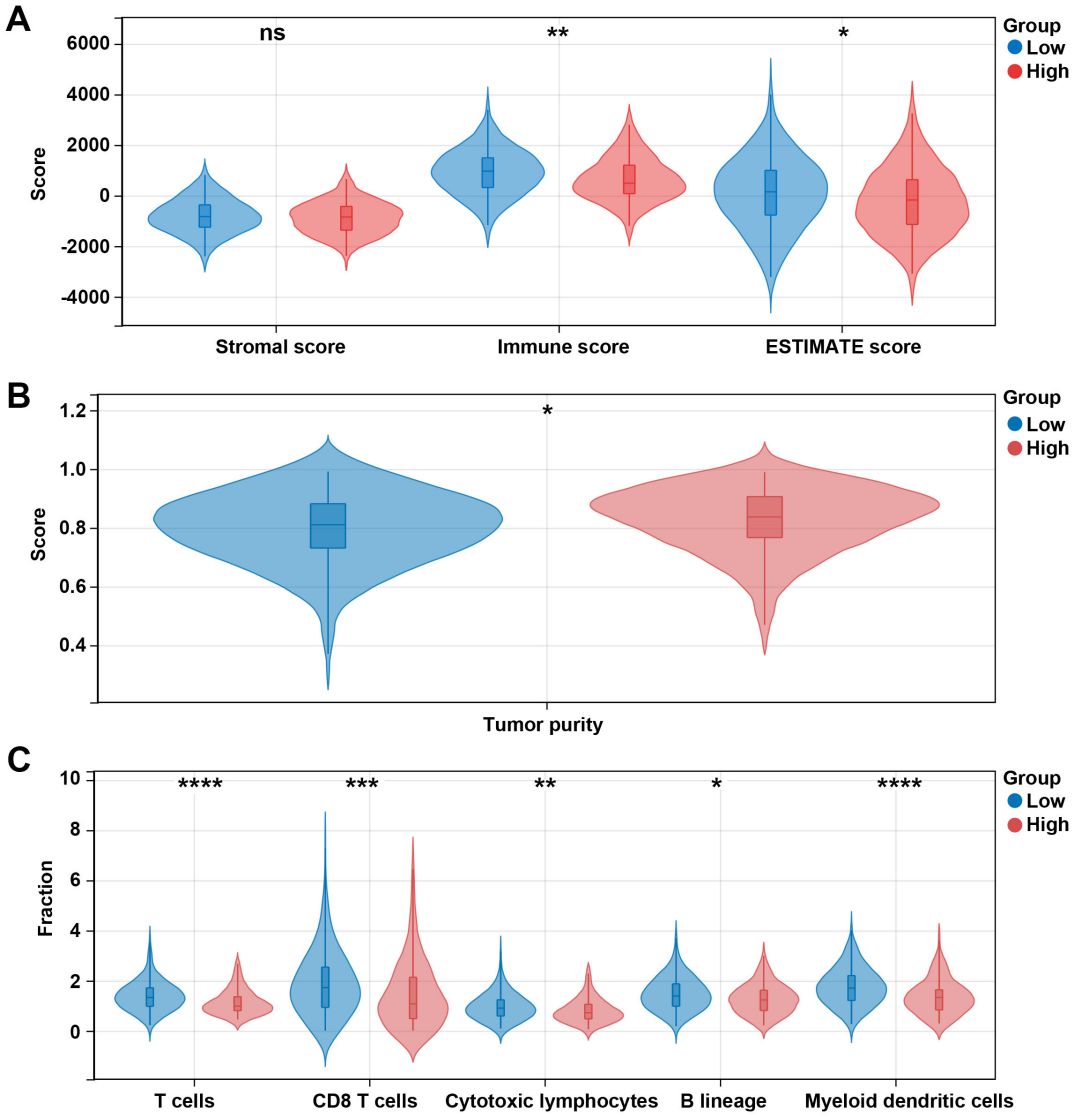
Different immune profiles between different risk groups in the TCGA dataset. **(A)** Stromal, immune, and ESTIMATE scores between the low- and high-risk groups. **(B)** Tumor purity between the low- and high-risk groups. **(C)** Cell fraction between the low- and high-risk groups. *p < 0.05, **p < 0.01, ***p < 0.001, ****p < 0.0001, ns, no significant.

Given the above results that differentially expressed prognosis-related genes were related to metabolic pathways and that DEGs in high risk vs. low risk were related to the immune system process, we then explore the immune microenvironment between different risk groups. Our data showed that immune and ESTIMATE scores in the low-risk group were significantly higher than those in the high-risk group (p < 0.05, **Figure 7A**), and the high-risk group had a higher tumor purity (**Figure 7B**). Additionally, we observed five immune cells were significantly infiltrated in the low-risk group, including T cells, CD8 T cells, cytotoxic lymphocytes, B lineage, and myeloid dendritic cells (**Figure 7C**).

**Figure 8:**
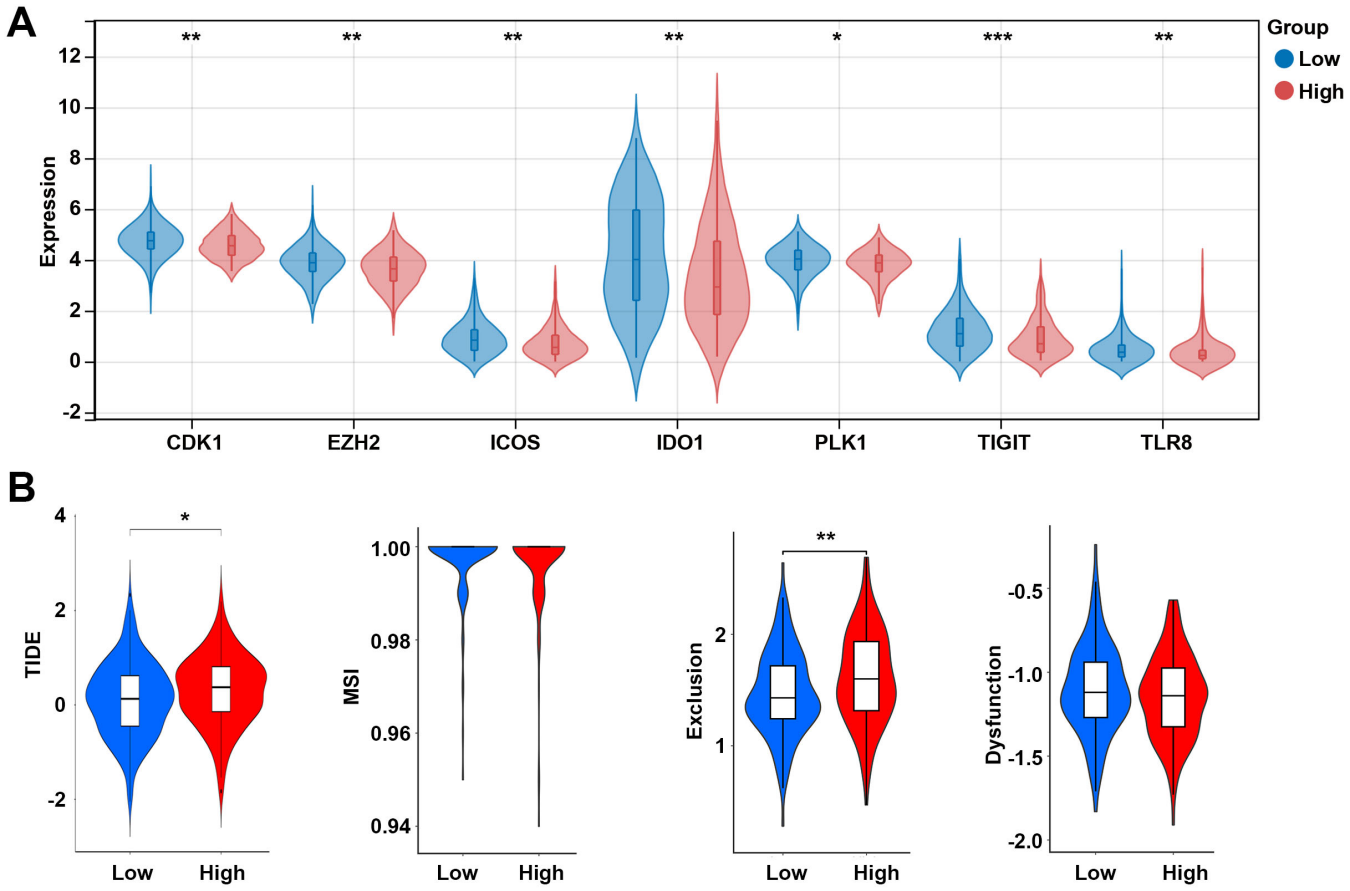
Analysis of immune checkpoints between the low- and high-risk groups. **(A)** Expression levels of immune checkpoint-related genes between the low- and high-risk groups. **(B)** TIDE score between the low- and high-risk groups. *p < 0.05, **p < 0.01, ***p < 0.001. TIDE, tumor immune dysfunction and exclusion; MSI, microsatellite instability.

The effector function of CD8 T cells is regulated by immune checkpoints. Due to the differences in the levels of CD8 T cells between high and low risk groups, we further analyzed the differences in immune checkpoint gene expression between high and low risk groups. Among 13 checkpoint-related genes, 7 genes were observed to be significantly correlated to the risk score, and all of them were downregulated in the high-risk group, including CDK1, EZH2, ICOS, IDO1, PLK1, TIGIT, and TLR8 (p < 0.05, **Figure 8A**). Further research showed that the high-risk group had a higher TIDE score and exclusion score than the low-risk group (**Figure 8B**). These results suggest that the high risk group is associated with an immunosuppressive environment.

### 3.6 Expression of hub genes

**Figure 9:**
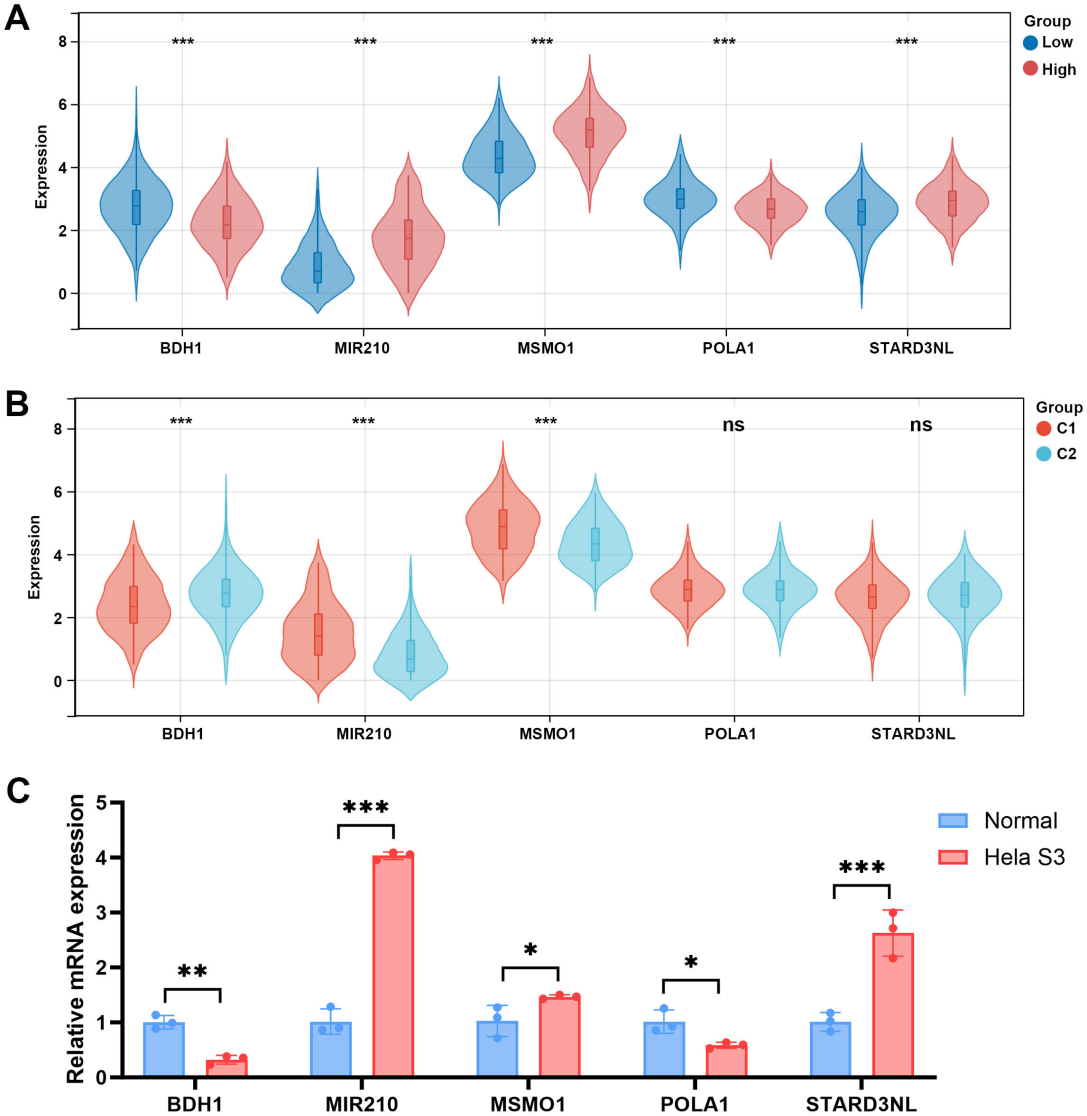
Expression levels of 5 MMRGs in different groups. **(A)** Expression levels of 5 prognostic genes in the low- and high-risk groups. **(B)** Expression levels of 5 prognostic genes in C1 and C2. **(C)** Expression levels of 5 prognostic genes in human cervical epithelial cells and CC cells. *p < 0.05, **p < 0.01, ***p < 0.001. MMRG, mitochondrial metabolism-related gene; CC, cervical cancer.

To further explore whether the differences in the microenvironment between different groups are related to the hub genes, we examined the expression of these hub genes among different groups. Compared to the low-risk group, BDH1 and POLA1 were downregulated in the high-risk group, while MIR210, MSMO1, and STARD3NL were upregulated (**Figure 9A**). Similarly, in the C1 group, BDH1 and POLA1 were downregulated, while MIR210, MSMO1, and STARD3NL were upregulated (**Figure 9B**). These results are consistent with previous findings, indicating a higher mortality risk and therefore a poorer prognosis in the C1 group. Furthermore, we conducted cell experiments to validate the expression of these genes. Compared to human cervical epithelial cells, BDH1 and POLA1 were downregulated, while MIR210, MSMO1, and STARD3NL were upregulated in the CC cell line Hela S3 cells (**Figure 9C**). These findings suggest that the expression of these five hub genes may be closely related to tumor immune-metabolic regulation.

### 3.7 Drug sensitivity analysis

The correlation between the expression levels of 5 prognostic genes and drug sensitivity was further investigated utilizing the CellMiner database. The top three drugs significantly correlated with the expression of each gene are shown in **Supplementary Figure S1**. BDH1 is positively associated with ciclosporin, Raloxifene, and Tamoxifen (**Supplementary Figure S1A**). MIR210 was positively associated with Cediranib, ergenyl, and Motesanib (**Supplementary Figure S1B**). Similarly, MSMO1 displayed a positive correlation with Amiodarone, uridin, and Zoledronate (**Supplementary Figure S1C**), and POLA1 was positively associated with Methylprednisolone, PX-316, and ZM-336372 (**Supplementary Figure S1D**). STARD3NL showed a positive association with JNJ-38877605 and Lovastatin while displaying a negative association with Fluorouracil (**Supplementary Figure S1E**). Among these drugs, the high-risk group was significantly sensitized to certain drugs, including ciclosporin, Raloxifene, Tamoxifen, Zoledronate, and Lovastatin (**Supplementary Figure S2**).

## 4 Discussion

Although advances in the treatment of CC have been made, its poor prognosis still poses a significant threat to women’s health (4). Recent studies suggest that MM is essential for tumor growth, and some clinical trials have demonstrated the feasibility of modulating MM to treat cancer (23). The bioinformatic research on MM in CC is limited. Therefore, this study constructed a prognostic model based on five MMRGs for CC and explored their association with the immune microenvironment.

Metabolic remodeling is one of the hallmarks of cancer. Current evidence indicates that MM-related pathways are reprogrammed in cancer, playing crucial roles in bioenergetics, biosynthesis, and redox homeostasis (24). The regulation of redox balance in tumor cells is influenced by their significantly increased glucose uptake, which produces TCA cycle metabolites. These metabolites supply electrons to the mitochondrial electron transport chain (ETC) (25). Inhibiting ETC-related genes could heighten the vulnerability of cancer cells to glucose depletion, consequently impeding tumor progression (26). MMRGs have been considered prognostic markers for various cancers, including breast cancer (27), osteosarcoma (28), and ovarian cancer (29). Based on the MMRGs. the present study identified two molecular subtypes of CC, and C1 showed shorter OS time than C2. Additionally, 5 key prognostic MMRGs in CC were identified, including BDH1, MIR210, MSMO1, POLA1, and STARD3NL. BDH1 is a key catalytic enzyme in ketogenesis, catalyzing the reversible conversion of acetoacetate to beta-hydroxybutyrate (30). Within the mitochondria, ketone bodies undergo oxidation via the TCA cycle, leading to the generation of acetyl-CoA and NADH. Downregulation of BDH1 is a prognostic marker in hepatocellular carcinoma (31). POLA1 encodes DNA polymerase, which facilitates DNA replication and repair, ensuring the maintenance of mitochondrial genome integrity. POLA1 has antitumor activity in inhibiting cancer cell proliferation and inducing apoptosis (32). A previous study demonstrates that POLA overexpression is associated with the poor prognosis of CC patients (33). MIR210 originates from mitochondria, its expression correlates with hypoxia gene signatures, and it could reduce the activity of proteins controlling MM (34). Nakada et al. points out that MIR210 induces energy metabolism shift from OXPHOS to glycolysis via acting on the mitochondrial inner membrane (35). MSMO1 catalyzes the demethylation of C4-methyl sterol, a critical step in cholesterol biosynthesis within mitochondria. Abnormal expression of MSMO1 would lead to CC (36). STARD3NL is involved in MM by mediating the transfer of cholesterol between membranes, potentially contributing to lipid metabolism and homeostasis within mitochondria (37). The risk score constructed using these five MMRGs exhibits good prognostic function for patients with CC. Patients within the low-risk group have longer survival time.

Furthermore, KEGG analysis of DEGs in the normal and tumor groups was enriched for several metabolic pathways. Overexpressed genes and downregulated genes in C1 were also associated with metabolic pathways. Metabolism and immunity are usually inextricably linked. After further analyzing the function of DEGs in high- and low-risk populations, we found that these DEGs are primarily associated with the immune system, including the Th17 cell differential, IL-17 signaling pathway, and MAPK signaling pathway. Th17 cells are a subset of T-helper cells that produce IL-17, a pro-inflammatory cytokine (38). IL-17 acts on tumor cells and various components of the tumor microenvironment to promote tumor growth, angiogenesis, and metastasis (39). It can also induce the production of other pro-inflammatory cytokines and chemokines, creating a pro-tumor inflammatory environment (40). The MAPK signaling pathway is a crucial intracellular signaling cascade involved in cell proliferation, survival, and differentiation (41). Activation of MAPK signaling can occur downstream of IL-17 receptor engagement. In breast cancer, the Th17/IL-17/MAPK cascade signaling pathway plays a multifaceted role in cancer progression (42). Previous studies have indicated that MM abnormalities can influence tumor cell antigen presentation and processing, thereby aiding tumor cells in evading recognition and attack by the immune system (43). Activation of the IL-17/MAPK signaling pathway can lead to an increase in immunosuppressive cells such as regulatory T cells and myeloid-derived suppressor cells (MDSCs), thereby inhibiting the anti-tumor immune response. Additionally, activation of the IL-17/MAPK signaling pathway may result in changes in tumor cell surface antigens, making tumor cells less recognizable and susceptible to clearance by the immune system (44). Th17 and the MAPK signaling pathway have been demonstrated to play a pro-econogenic role in promoting CC (45, 46). Therefore, we hypothesize that MMRGs may enhance tumor immune evasion by modulating the IL-17/MAPK signaling pathway in cervical cancer.

Given the crucial role of the immune microenvironment in cancer progression, we further investigated differences in immune cell infiltration and immune scores among individuals at different risk groups. The results indicated that individuals at low risk had higher immune scores, and correspondingly, we observed more immune cell infiltration in the low-risk group, including T cells, CD8 T cells, cytotoxic lymphocytes, B lineage cells, and myeloid dendritic cells. These findings demonstrate the correlation between MM-related prognostic models and immune infiltration in CC. To regulate immune responses, PD-L1 was expressed in these immune cells to maintain immune homeostasis and protect the body from foreign pathogens (47). Furthermore, analysis of immune checkpoint-related genes revealed higher expression of immune checkpoints in the low-risk group, suggesting better efficacy of immune therapy in this group compared to the high-risk group. Among the identified immune checkpoint genes, TIGIT showed the most significant difference between the two groups. TIGIT is a crucial target in tumor immunotherapy, as it can prevent NK cells from releasing tumor antigens, impair dendritic cell-induced T cell priming, or inhibit CD8+ T cell-mediated killing of cancer cells (48). This is consistent with the findings of Han et al., suggesting that TIGIT may kill cancer cells in the low-risk group by reducing dendritic cell-triggered T-cell priming (49). However, the specific mechanism of action of TIGIT in cervical cancer remains to be further elucidated. Additionally, Our results revealed that BDH1 expression is positively related to ciclosporin, a typical immunosuppressive drug with an anti-tumor effect (50). Importantly, the drug sensitivity of ciclosporin is higher in the high-risk group, indicating that CC patients have better outcomes with this drug. Research suggests that alterations in MM within cancer cells may also modulate drug sensitivity and resistance through the MAPK signaling pathway (51). Whether MM-modulated MAPK signaling influences the drug sensitivity of ciclosporin needs further research.

## 5 Conclusion

In conclusion, this study elucidated the significance of MM in CC progression and its interplay with the immune microenvironment. By constructing a prognostic model based on five MMRGs (BDH1, MIR210, MSMO1, POLA1, and STARD3NL) and exploring their association with immune infiltration, significant insights were gained into CC treatment. Furthermore, the study highlighted the potential involvement of the Th17/IL-17/MAPK signaling pathway in mediating immune escape in CC, possibly influenced by MMRGs. Additionally, the analysis of immune checkpoint-related genes suggested a potential for improved efficacy of immune therapy in the low-risk group, with TIGIT emerging as a significant target. These findings underscore the intricate relationship between MM, immune regulation, and therapeutic outcomes in CC, providing valuable insights for the development of novel prognostic markers and therapeutic strategies.

## Data Availability

The raw data supporting the conclusions of this article will be made available by the authors, without undue reservation.

## Supplementary material

Supplementary Figure 1 Correlation between predicted drugs and prognostic genes. **(A)** Correlation of BDH1 with ciclosporin, Raloxifene, and Tamoxifen. **(B)** Correlation of MIR210 with Cediranib, ergenyl, and Motesanib. **(C)** Correlation of MSMO1 with Amiodarone, uridin, and Zoledronate. **(D)** Correlation of POLA1 with Methylprednisolone, PX-316, and ZM-336372. **(E)** Correlation of STARD3NL with JNJ-38877605, Lovastatin, and Fluorouracil.

Supplementary Figure 2 Drug sensitivity between different risk groups. *p < 0.05, **p < 0.01, ns, no significant.
