# Supplementary figures and images for: Bioinformatics and experimental approach reveal potential prognostic and immunological roles of key mitochondrial metabolism-related genes in cervical cancer
Source: Front Oncol. 2025 Mar 17;15:1522910. doi: 10.3389/fonc.2025.1522910 (PMC11955473; doi:10.3389/fonc.2025.1522910)

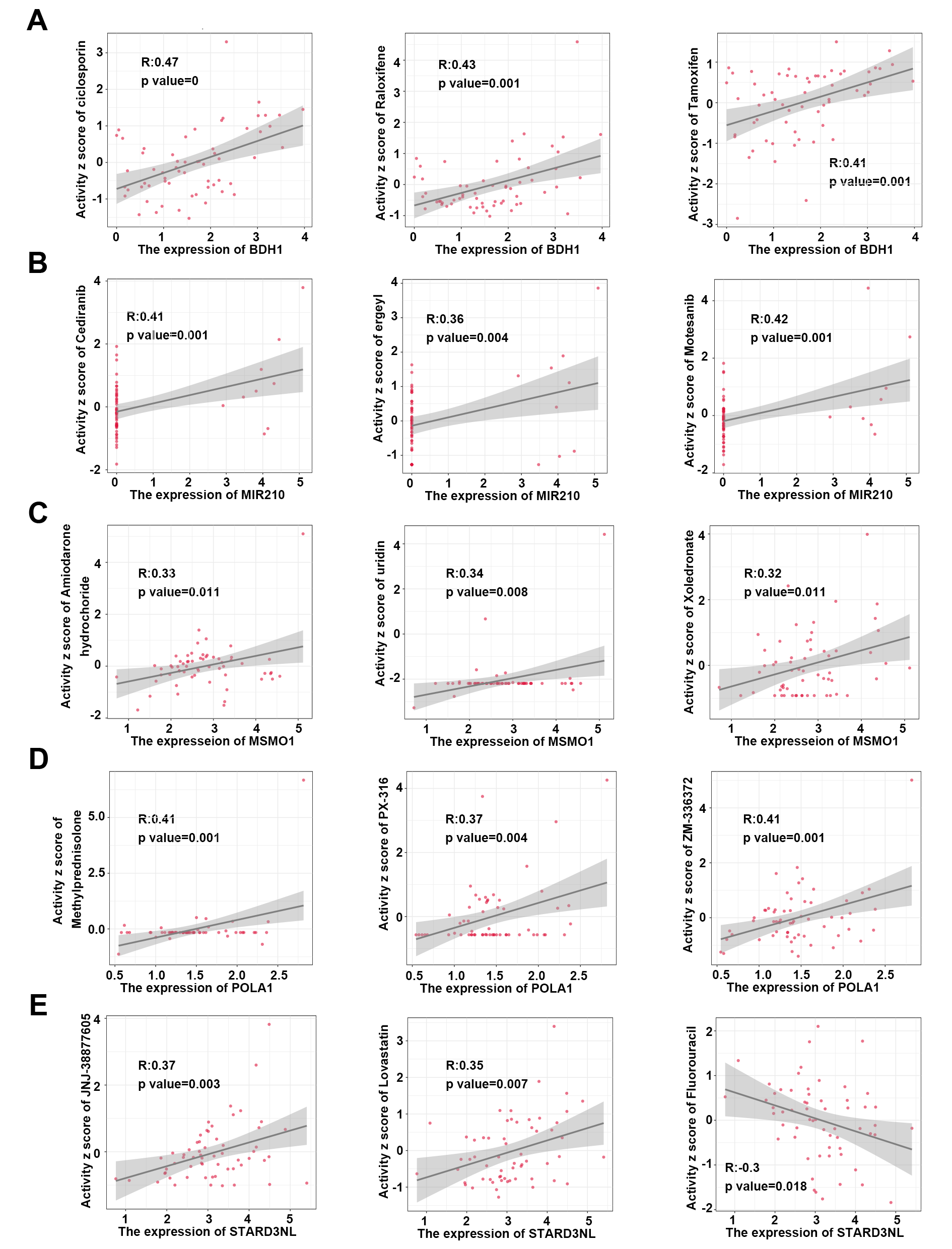

Supplement: Supplementary Figure 1 — Correlation between predicted drugs and prognostic genes. (A) Correlation of BDH1 with ciclosporin, Raloxifene, and Tamoxifen. (B) Correlation of MIR210 with Cediranib, ergenyl, and Motesanib. (C) Correlation of MSMO1 with Amiodarone, uridin, and Zoledronate. (D) Correlation of POLA1 with Methylprednisolone, PX-316, and ZM-336372. (E) Correlation of STARD3NL with JNJ-38877605, Lovastatin, and Fluorouracil. [file Image1.tif]

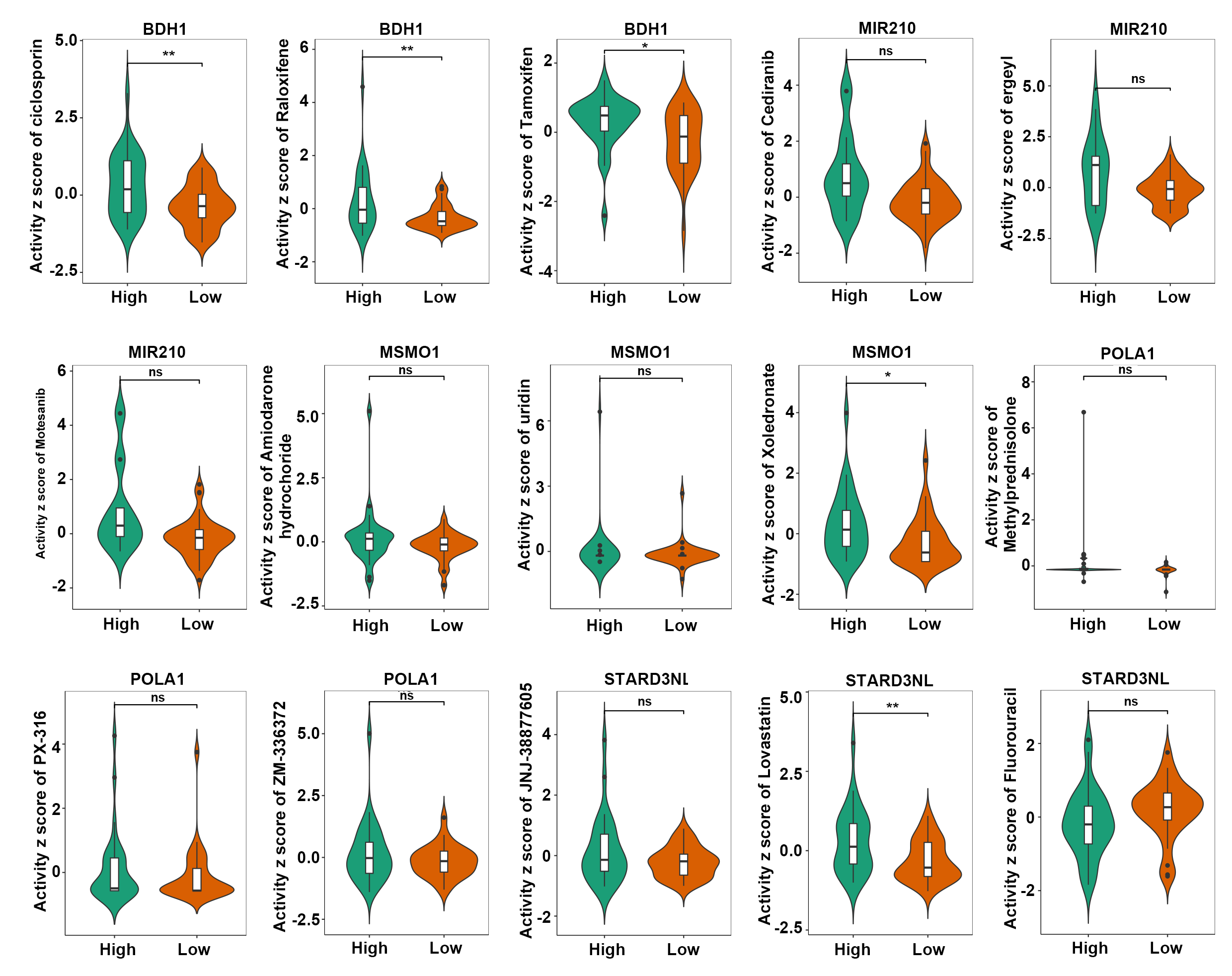

Supplement: Supplementary Figure 2 — Drug sensitivity between different risk groups. *p < 0.05, **p < 0.01, ns, no significant. [file Image2.tif]
