# Supplementary material for: Bioinformatics and experimental approach reveal potential prognostic and immunological roles of key mitochondrial metabolism-related genes in cervical cancer
Source: Front Oncol. 2025 Mar 17;15:1522910. doi: 10.3389/fonc.2025.1522910 (PMC11955473; doi:10.3389/fonc.2025.1522910)
Supplement: Supplementary file 3 [file Table1.docx]

**Table S1 Primer sequences in RT-qPCR**

| Genes | Sequences |
| --- | --- |
| BDH1-forward | ACTGGTACCTCTGCATGTCC |
| BDH1-reverse | CTCCATACAGCCAGCTCTGA |
| POLA1-forward | GTCGTGATGGCTTTCAGCAT |
| POLA1-reverse | TGTGACTGAAAGGGAGGCTT |
| MSMO1-forward | GGGTGACCATTCGTTTAT |
| MSMO1-reverse | AAATTCGATCCCACCATG |
| STARD3NL-forward | GCTATGTGCTGCCCATCATT |
| STARD3NL-reverse | CCCTCTCTGAAGCATCCTGA |
| MIR210-forward | ACACTCCAGCTGGGAGCCCCTGCCCACCGC |
| MIR210-reverse | TGGTGTCGTGGAGTCG |
| GAPDH-forward | GGAGCGAGATCCCTCCAAAAT |
| GAPDH-reverse | GGCTGTTGTCATACTTCTCATGG |
